# Programmable (proSA®) vs. fixed (SHUNTASSISTANT®) gravitational valves in pediatric patients with hydrocephalus: a 16-year retrospective single-center comparative study with biomechanical analysis

**DOI:** 10.1007/s00701-023-05751-y

**Published:** 2023-08-29

**Authors:** Mohammed Issa, Filippo Paggetti, August von Hardenberg, Christoph Miethke, Andreas W. Unterberg, Ahmed El Damaty

**Affiliations:** 1 Department of Neurosurgery, Heidelberg University Hospital, Im Neuenheimer Feld 400, 69120 Heidelberg, Germany; 2 Christoph Miethke GmbH & Co. KG, Potsdam, Germany

**Keywords:** Hydrocephalus, proSA®, SHUNTASSISTANT®, Gravitational valve, Overdrainage, Underdrainage

## Abstract

**Purpose:**

In pediatric hydrocephalus (HC) treatment, programmable gravitational valves offer greater flexibility to manage overdrainage during children’s growth. However, it remains unclear whether these devices provide better outcomes rather than their precursors. The study assessed the benefit from programmability of gravitational valve, i.e., programmable-SHUNTASSISTANT (proSA®) vs. SHUNTASSISTANT® (SA®).

**Methods:**

Clinical records and imaging of pediatric patients with hydrocephalus of non-tumoral etiology treated with fixed (SA®) or programmable (proSA®) gravitational valves between January 2006 and January 2022 were analyzed in a retrospective single-center study. Valve survival was compared in relation to age and etiology. Lately explanted valves received biomechanical analysis.

**Results:**

A total of 391 gravitational valves (254 SA® and 137 proSA®) were inserted in 244 patients (*n* = 134 males). One hundred thirty-three SA® (52.4%) and 67 proSA® (48.9%) were explanted during a follow-up of 81.1 ± 46.3 months. Valve survival rate at 1 and 5 years with proSA® was 87.6% and 60.6% compared to 81.9% and 58.7% with SA®, with mean survival time 56.4 ± 35.01 and 51.4 ± 43.0 months, respectively (*P* = 0.245). Age < 2 years at implantation correlated with significantly lower valve survival rates (*P* < 0.001), while HC etiology showed no significant impact. Overdrainage alone accounted for more SA® revisions (39.8% vs. 3.1%, ***P*** < 0.001), while dysfunctions of the adjustment system represented the first cause of valve replacement in proSA® cohort (45.3%). The biomechanical analysis performed on 41 proSA® and 31 SA® showed deposits on the valve’s internal surface in 97.6% and 90.3% of cases.

**Conclusion:**

Our comparative study between proSA® and SA® valves in pediatric HC demonstrated that both valves showed similar survival rates, regardless of etiology but only with young age at implantation. The programmability may be beneficial in preventing sequelae of chronic overdrainage but does not reduce need for valve revision and proSA® valve should be considered in selected cases in growing children older than 2 years.

## Introduction

Ventriculoperitoneal (VP) shunting is a common treatment for hydrocephalus (HC) in children [15]. However, associated complications can result in frequent revisions, impacting a child’s quality of life and development [7, 13, 14]. Chronic overdrainage of cerebrospinal fluid (CSF) after VP shunt is a challenging complication that can contribute to clinical deterioration [7, 13, 22, 23, 29], and the economic burden of repeated valve replacements [26]. A “siphon effect” created by gravity and hydrostatic force when the patient is upright causes overdrainage [10]. This can lead to postural headaches, as well as subdural hematoma/hygromas, slit-ventricle syndrome, and secondary craniosynostosis. It also predisposes the patient to other mechanical complications, such as ventricular catheter occlusion [7, 20].

The primary cause of overdrainage after VP shunt implantation is the effect of posture [20], and numerous overdrainage-preventing devices have been developed on that principle [14]. Gravitational valves (e.g., Miethke SHUNTASSISTANT, SA®) are antisiphons implanted in tandem with standard differential pressure (DP) valves to prevent postural overdrainage. Their combination with adjustable DP valves also addresses other predisposing factors, e.g., anatomical circumstances, physical activity, and CSF imbalance [9]. Throughout the process of growth, alterations in these factors can lead to the progression of excessive drainage even after adjustments are made to the DP valve. As a result, it may be necessary to implant a new gravitational valve that has a higher pressure threshold to address the issue [8, 14]. The introduction of more advanced programmable gravitational valves (e.g., Miethke programmable-SHUNTASSISTANT, proSA®) enabled the non-invasive tuning of the pressure settings, improving the compliance of the antisiphon device. Nonetheless, the benefits of programmability may be undermined by new mechanical complications, such as blockage of the valve adjustment system by protein deposits [17], and unintentional changes in the pressure setting. Therefore, selecting the most suitable device for pediatric HC patients is crucial to manage overdrainage effectively and minimize the need for additional revisions.

We aimed to evaluate the advantages of utilizing programmable gravitational valves (proSA® versus SA®) in our pediatric patients and to identify the risk factors that contribute to frequent valve dysfunctions, taking into account etiological and age-related differences. Gaining a deeper understanding of these risk factors would permit a more personalized approach to the use of these valves, resulting in a more favorable cost-benefit ratio while reducing the need for additional valve revisions and associated risks to the patients.

## Methods

### Study design and patient sample

In a retrospective single-center study, we assessed efficacy of fixed (SA®) and programmable (proSA®) gravitational valves in treating pediatric hydrocephalus patients. We included all patients harboring ventriculoperitoneal or ventriculoatrial shunts with a minimum follow-up of 12 months after implantation between January 2006 and January 2022. All parents or patients’ guardians signed informed consent for participation in that study. The institutional ethics committee approved the study (S-084/2022); we excluded patients with tumor-related hydrocephalus as their survival depends on oncological disease and they are more prone to valve dysfunction due to possible presence of tumor cells in their CSF. Primary outcome was to compare survival rates of the two valves using Kaplan-Meier survival analysis and number of shunt revisions. Secondary objective was to identify potential risk factors for valve dysfunction by subgroup analysis according to etiology (posthemorrhagic, aqueduct stenosis, and Chiari malformation type II) and age (0–2, 2–10, and 10–18 years) groups, facilitating informed decisions when selecting valves for the treatment of pediatric hydrocephalus patients. Since 2017, all patients under the age of 6 months and all those who need reoperation on their shunt due to CSF infection received an antibacterial impregnated catheter. Before 2017, the indication for such catheters was only made individually.

Infection in our study included CSF, wound, and peritoneal infections. Each infection was determined individually according to established criteria. CSF was examined in the case of CSF infection, microbiological material was examined in the case of wound infection, and microbiological material was surgically obtained in the case of abdominal infection. For all types of infection, proof of pathogens was required.

### Clinical and radiological parameters

Data about patients’ demographics, HC etiology, shunt/valve insertion indication, pre-/postoperative clinical and radiological assessments, frequency of pressure settings adjustments, and shunt revisions were collected from our central database. Valves explanted starting from mid-2017 were sent to the manufacturer’s laboratory (MIETHKE) for biomechanical analysis. Pederesen et al. defined overdrainage as the persistency of clinical symptoms and signs as postural dependent headache and vomiting, sunken fontanelle, decreasing head circumference and/or radiological signs (slit ventricles and/or subdural collections) [21]. Good clinical outcome was defined as stable improvement of the preoperative symptoms within the first postoperative year, not requiring valve explantation. The evaluation of the postoperative correction of chronic signs of overdrainage, such as arrested head growth, was conducted during both short-term and long-term follow-up periods. The objective was to ensure continued head growth aligned with the initial percentile curve. Patients with preoperative slit-ventricle morphology were evaluated to determine the radiological outcome. A normalized ventricular size following surgery was considered an improved radiological finding.

### Biochemical analysis of explanted valves

The biomechanical analysis assessed macroscopic changes or deformations in the valve casing, valve permeability, the flow resistance in cm H_2_O during horizontal and vertical positioning of the device, adjustability in vitro of the proSA® valves, and the presence of internal deposits. For proSA® valves, we classified the internal deposits into three grades according to their extension on the valve’s inner surface: grade 1 (< 25%), grade 2 (25–50%), and grade 3 (> 50%). Two observers rated each valve separately, with coincidental results.

### Data analysis

Values were reported as mean ± standard deviation for continuous variables and as frequencies and percentages for categorical variables. For intergroup comparisons, t-test was utilized to compute differences between continuous variables, and Mann-Whitney test and Fisher’s exact test for categorical variables. A *P*-value less than 0.05 indicated statistical significance. IBM SPSS Statistics 28 software was used for graph design and statistical analysis.

## Results

### Patients’ population

**Fig. 1:**
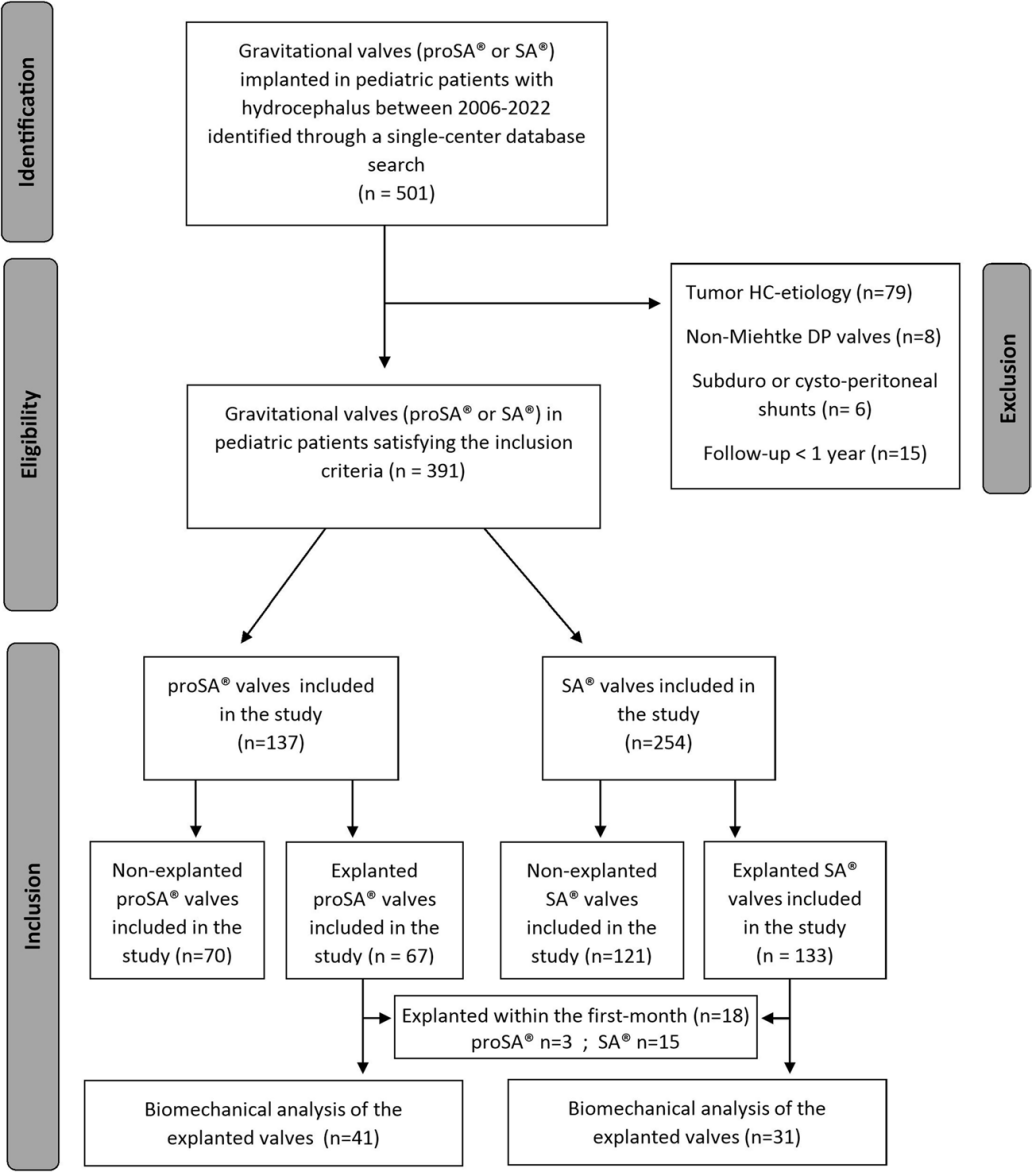
Flow diagram illustrating the process of inclusion and exclusion of the study cohort

Three-hundred ninty-one gravitational valves (254 SA®, 137 proSA®) inserted in 244 pediatric patients (134 males) were included in the study. Figure 1 shows the inclusion and exclusion process in detail. The mean age at the time of valve insertion was 5.51 ± 5.15 years in the proSA® cohort and 5.8 ± 5.52 in the SA® cohort (*P* = 0.608), while the mean time of follow-up was 78.19 ± 30.19 and 82.70 ± 52.96 months, respectively (*P* = 0.284). (See Table 1.)

**Table 1:** Cases characteristics and valve revisions*

	Total	proSA® group	SA® group	*P*-value**
Number of valves	373	134 (35.9)	239 (64.1)	
Gender				
Male	203 (54.4)	77 (57.5)	126 (52.7)	0.388
Female	170 (45.6)	57 (42.5)	113 (47.3)	
Mean age (years) ***	5.95 ± 5.39	5.63 ± 5.15	6.13 ± 5.52	0.379
Follow-up (months) ***	84.92 ± 43.95	79.93 ± 28.16	87.71 ± 50.54	0.057
Type of shunt				0.717
Ventriculoperitoneal	356 (95.4)	127 (94.8)	229 (95.8)	
Ventriculoatrial	17 (4.6)	7 (5.2)	10 (4.2)	
Differential pressure (DP) valve in the shunt system				** < 0.001**
New DP valve implanted with proSA® or SA®	302 (81.0)	93 (69.4)	209 (87.4)	
Previous DP valve maintained with new proSA® or SA®	71 (19.0)	41 (30.6)	30 (12.6)	
Type of DP valves				** < 0.001**
Adjustable DP valves	363 (97.3)	126 (94.0)	237 (99.2)	
proGAV®	212 (58.4)	68 (54.0)	144 (60.8)	
proGAV® 2.0	151 (41.6)	58 (46.0)	93 (39.2)	
Fixed DP valves	10 (2.7)	8 (6.0)	2 (0.8)	
miniNAV®	8 (80.0)	8 (100)	0	
paediGAV®	2 (20.0)	0	2 (100)	
Hydrocephalus etiologies				0.902
Post-hemorrhagic	166 (44.5)	56 (41.8)	110 (46.0)	
Idiopathic aqueduct stenosis	63 (16.9)	21 (15.7)	42 (17.6)	
Chiari malformation type II	59 (15.8)	24 (17.9)	35 (14.6)	
Postinfectious	18 (4.8)	7 (5.2)	11 (4.6)	
Arachnoidal cyst	22 (5.9)	7 (5.2)	15 (6.3)	
Others	45 (12.1)	19 (14.2)	26 (10.9)	
Indication for proSA® and SA® valve implantation				0.201
Primary valve implantation	80 (21.4)	24 (17.9)	56 (23.4)	
Post-hemorrhagic hydrocephalus	31 (38.8)	10 (41.7)	21 (37.5)	
Idiopathic aqueduct stenosis	16 (20.0)	1 (4.2)	15 (26.8)	
Chiari malformation type II	11 (13.8)	7 (29.2)	4 (7.1)	
Others	22 (27.5)	6 (25.0)	16 (28.6)	
Secondary valve implantation	293 (78.6)	110 (82.1)	183 (76.6)	
Adjustment difficulties	90 (30.7)	30 (27.3)	60 (32.8)	
Overdrainage	86 (29.4)	41 (37.3	45 (24.6)	
Underdrainage	35 (11.9)	12 (10.9)	23 (12.6)	
Associated with catheter revision	47 (16.0)	14 (12.7)	33 (18.0)	
Post-CSF infection	24 (8.2)	7 (6.4)	17 (9.3)	
Others	11 (3.8)	6 (5.5)	5 (2.7)	
Explanted gravitational valves	182 (48.8)	64 (47.8)	118 (49.4)	0.849
Indication for proSA® or SA® valve explantation				** < 0.001**
Gravitational valve adjustment difficulties	29 (15.9)	29 (45.3)	0	
DP valve adjustment difficulties	36 (19.8)	5 (7.8)	31 (26.3)	
Overdrainage	51 (28.0)	2 (3.1)	49 (41.5)	
Underdrainage	25 (13.7)	12 (18.8)	13 (11.0)	
Catheter-related causes	23 (12.6)	9 (14.1)	14 (11.9)	
Infection	14 (7.7)	6 (9.4)	8 (6.8)	
Others	4 (2.2)	1 (1.6)	3 (2.5)	
Shunt systems requiring revisions without proSA® or SA® explantation;	128 (34.3)	55 (41.0)	73 (30.5)	0.053
Total revision	232	104	128	
Wound revision	32 (13.8)	19 (8.3)	13 (10.2)	
Operative function testing	18 (7.8)	8 (7.7)	10 (7.8)	
Proximal catheter dislocation or occlusion	87 (37.5)	32 (30.8)	55 (43.0)	
Distal catheter dislocation or occlusion	47 (20.3)	25 (24.0)	22 (17.2)	
Distal abdominal infection	9 (3.9)	3 (2.9)	6 (4.7)	
DP valve replacement	39 (16.8)	17 (16.3)	22 (17.2)	

(%) Data in parenthesis are percentages

^*^Without the early wound complications group

^**^Bold denotes statistical significance

^***^Data are given as mean ± standard deviation

### Early wound complications

After valve implantation, within the first month, a total of eighteen valves (4.6%) were replaced due to wound infections and healing disorders. Among these replaced valves, 3 were proSA® and 15 were SA® valves. Notably, all these implantations were performed in infants, with a median age of 0.27 years and a range of 0.0 to 1.0 years, exposing them to a higher risk of wound complications. None of those valves underwent biomechanical analysis by the manufacturer.

It is worth mentioning that only 38.9% of the explanted valves were as primary implants.

### Valve data and revisions

The valves were inserted as first implantations in 19% and 24% of cases. The secondary implanted proSA® and SA® replaced a previous gravitational valve in 70.0% and 64.5% of the cases and a non-gravitational device in 30.0% and 35.5%. Among these cases, the indication to shunt revision was mainly given by the insufficient control of overdrainage symptoms and signs with the previous shunt system (29.4%), thus requiring the implantation or substitution of a gravitational valve with a higher opening pressure. A further frequent indication was represented by the defective pressure adjustments of the previous system (30.7%), consisting of impossible or repeated accidental adjustments with inadequate CSF control. Hence, the system was replaced to address patients’ over- or underdrainage-related symptoms/signs and prevent possible complications.

During follow-up and after the first month of implantation, 168 and 246 shunt revisions were performed in proSA® and SA® cohorts. Sixty-four proSA® (47.8%) and 118 SA® (49.4%) valves were explanted during the analyzed period (*P* = 0.849). In proSA® cohort, dysfunctions of the adjustment system represented the first cause of valve replacement (45.3%), followed by underdrainage (18.8%). Valve explantations related to adjustment system dysfunction were performed either to prevent or to treat the onset of over- and underdrainage manifestations resulting from this defect. Overdrainage alone without adjustment system dysfunction accounted for only 3.1% of proSA® valve revisions, while it represented the most common cause of valve replacement in the SA® cohort (41.5%). Indications for valve explantations were significantly different among the two groups (*P* < 0.001). (For more details, see Table 1.)

### Comparative survival analysis

**Fig. 2:**
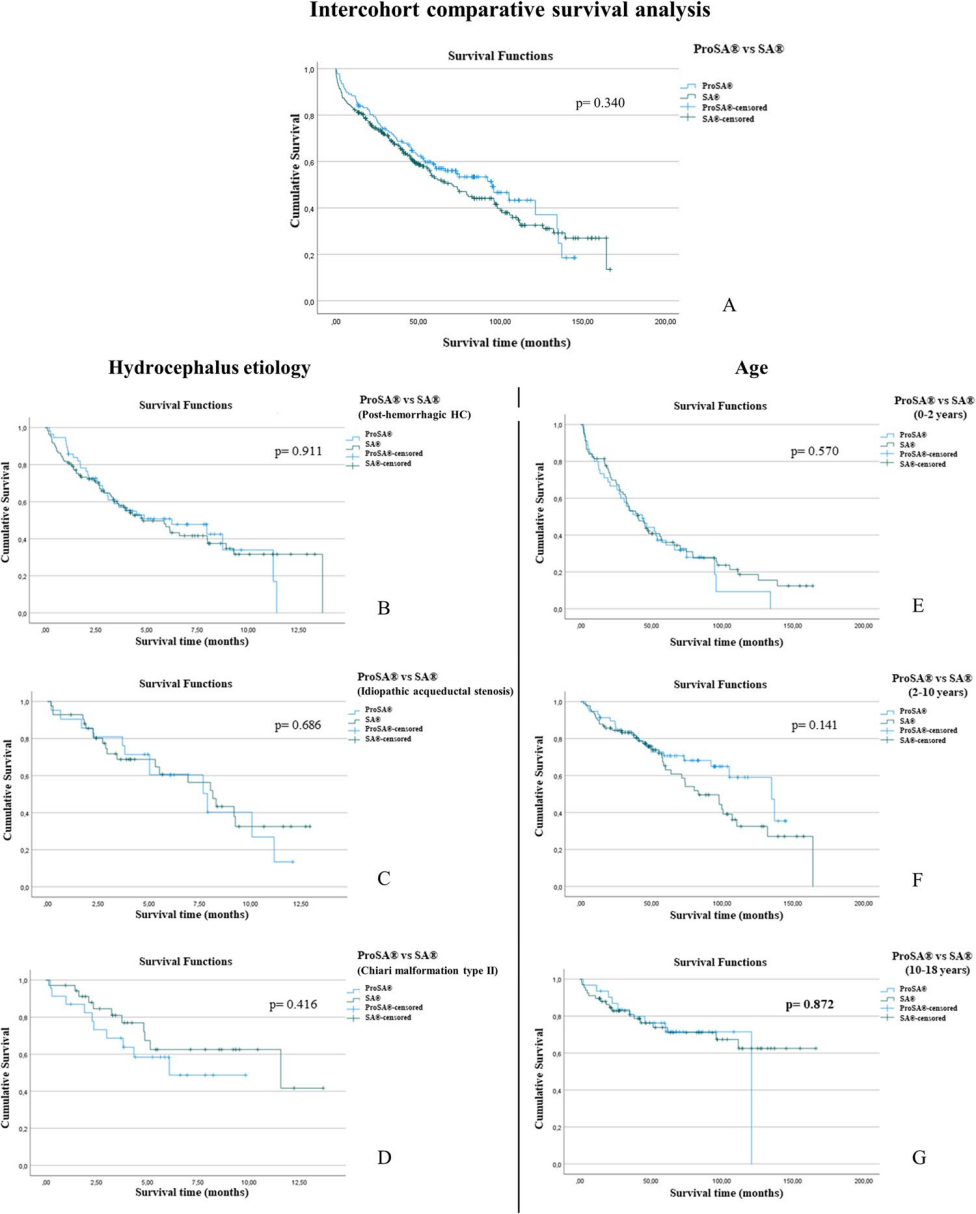
Intercohort analysis with Kaplan-Meier survival curves comparing proSA® and SA® valves. Curve A compares the entire proSA® and SA® cohorts while B, C, D, and E, F, and G compare the two cohorts under etiological and age subgroups, respectively. Etiological subgroups include posthemorrhagic (**A**), idiopathic aqueductal stenosis (**B**), and Chiari malformation type II (**C**). Age subgroups include 0–2 (**E**), 2–10 (**F**), and 10–18 (**G**) years

**Fig. 3:**
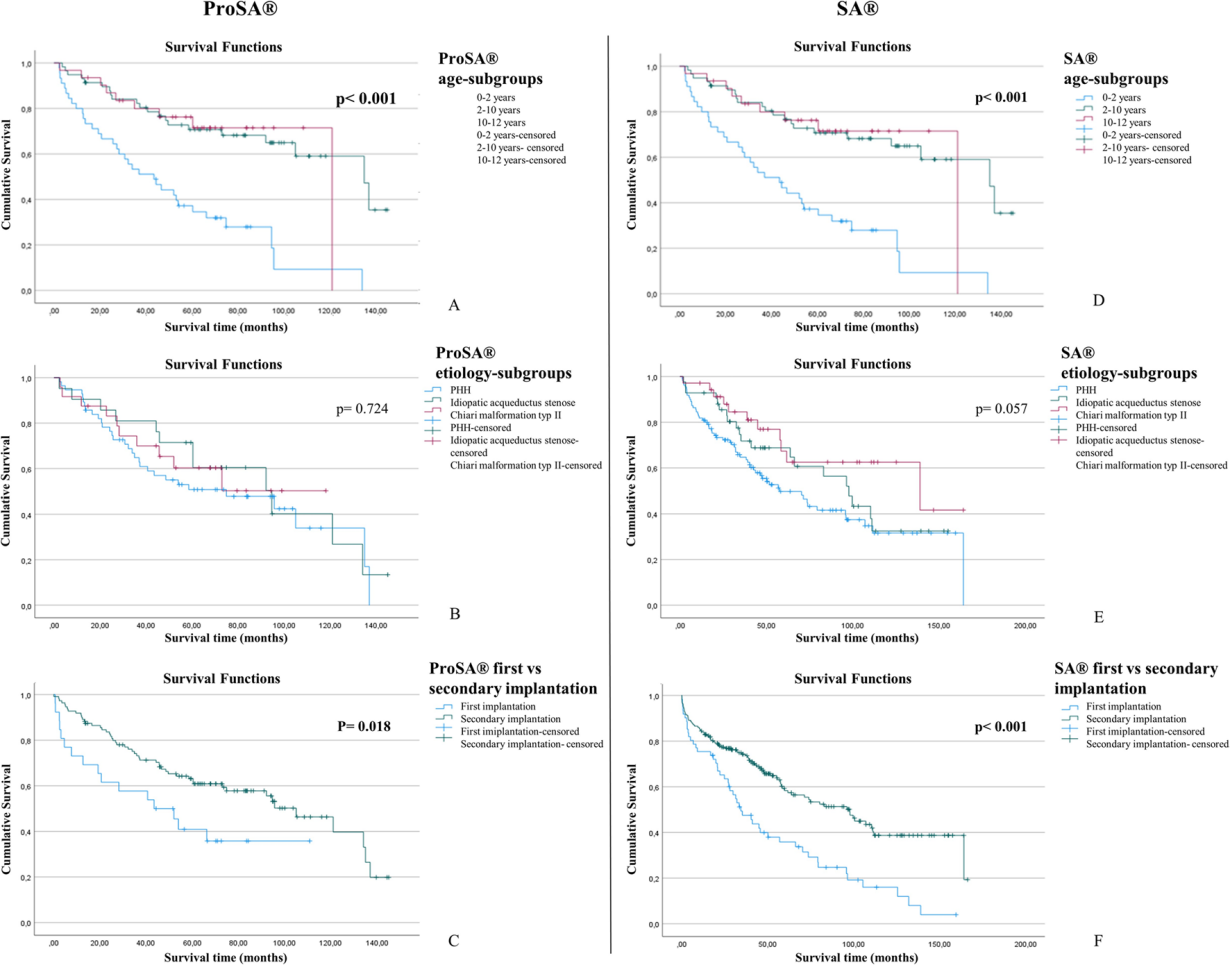
Intracohort comparative analysis with Kaplan-Meier survival curves evaluating the proSA® and SA® valves separately about age (**A**; **D**), hydrocephalus etiology (**B**; **E**), and primary vs. secondary implantations (**C**; **F**). Curves A, B, and C refer to proSA®, and curves E, F, and G refer to SA® valves

Valve survival rates at 1 and 5 years with proSA® were 87.6% and 60.6% compared to 81.9% and 58.7% with SA®, while the mean survival time was 56.4 ± 35.01 and 51.4 ± 43.0 months, respectively. No statistically significant difference was found in the overall valve survival rate between the two groups (*P* = 0.245). Inter- and intra-cohort Kaplan-Meier valve survival curves are shown in Figs. 2 and 3, respectively. Explanted and not explanted valves are compared in Table 2. Table 3 reports valve survival rates per year.

**Table 2:** Comparative analysis among proSA® and SA® explanted vs. non-explanted valves*

	proSA® valves			SA® valves		
	Not explanted	Explanted	*P*-value**	Not explanted	Explanted	*P*-value**
Number	70 (52.2)	64 (47.8)		121 (50.6)	118 (49.4)	
Age in years***	7.4 ± 5.2	3.7 ± 4.4	** < 0.001**	8.0 ± 5.4	4.2 ± 4.9	** < 0.001**
Male gender	42 (60.0)	35 (54.7)	0.601	42 (34.7)	35 (29.7)	0.601
Survival time in months***	74.5 ± 28.5	39.2 ± 34.9	** < 0.001**	67.6 ± 43.8	41.2 ± 36.1	** < 0.001**
Initial as the first implantation**	10 (14.3)	14 (21.9)	0.269	13 (10.7)	43 (36.4)	** < 0.001**
proSA adjustments in total**	2.1 ± 2.3	1.6 ± 1.8	0.177			
proSA® with adjustments difficulties	3 (4.3)	27 (42.2)	** < 0.001**			
proSA® with spontaneous adjustments	16 (22.9)	20 (31.3)	0.331			
DP adjustments in total	1.5 ± 1.5	1.3 ± 1.4	0.454	1.4 ± 1.6	1.8 ± 1.6	0.060
DP with adjustments difficulties	0.1 ± 0.3	0.3 ± 0.5	**0.002**	0.1 ± 0.3	0.4 ± 0.6	** < 0.001**
DP valve type						** < 0.001**
proGAV®	37 (52.9)	31 (48.4)		56 (46.3)	88 (74.6)	
proGAV® 2.0	29 (41.4)	29 (45.3)		65 (53.7)	28 (23.7)	
miniNAV®	4 (5.7)	4 (6.3)	0.922	0	0	
paediGAV®	0	0		0	2 (1.7)	
Etiology subgroups						0.073
Post-hemorrhagic HC	25 (35.7)	31 (48.4)		51 (42.1)	59 (48.8)	
Idiopathic aqueduct stenosis	9 (12.9)	12 (18.8)	0.472	22 (18.2)	20 (16.5)	
*Chiari malformation type II	14 (20)	10 (15.6)		24 (19.8)	11 (9.1)	
Slit ventricle in preoperative MRI	37 (52.9)	22 (34.4)	**0.037**	46 (38.0)	31 (26.3)	0.054

(%) Data in parenthesis are percentages

^*^Without the early wound complications group

^**^Bold denotes statistical significance

^***^Data are given as mean ± standard deviation

**Table 3:** proSA® and SA® valve survival rates

Variable	Valve	No. of pts	% valve survival								*P* value
			12 months	24 months	36 months	48 months	60 months	72 months	84 months	Max observation period (months)	
Total	proSA®	137	87.6	79.6	70.8	65.0	60.6	58.4	56.9	51.1 (145)	NS
	SA®	254	81.9	74.8	68.9	62.6	58.7	56.7	53.9	47.6 (166)	
0–2 years	proSA®	48	75.0	62.5	50.0	41.7	35.4	31.3	29.2	22.9 (134)	NS
	SA®	96	68.8	59.4	45.8	36.5	33.3	31.3	28.1	21.9 (164)	
2–10 years	proSA®	58	94.8	89.7	82.8	77.6	72.4	72.4	70.7	63.8 (145)	NS
	SA®	91	90.1	84.6	83.5	78.0	71.4	69.2	64.8	56.0 (164)	
10–18 years	proSA®	31	93.5	87.1	80.6	77.4	77.4	74.2	74.2	71.0 (121)	NS
	SA®	67	89.6	83.6	82.1	79.1	77.6	76.1	76.1	73.1 (166)	
PHH	proSA®	56	94.6	78.6	66.1	58.9	53.6	53.6	51.8	44.6 (137)	NS
	SA®	110	81.8	73.6	66.4	59.1	55.5	53.6	50.9	46.4 (164)	
IAS	proSA®	21	90.5	85.7	81.0	71.4	71.4	61.9	61.9	42.9 (145)	NS
	SA®	42	92.9	85.7	73.8	71.4	71.4	66.7	64.3	52.4 (155)	
Chiari II	proSA®	24	87.5	83.3	70.8	66.7	62.5	62.5	58.3	58.3 (118)	NS
	SA®	35	97.1	91.4	85.7	80.0	74.3	71.4	71.4	68.6 (164)	
Primary implant	proSA®	26	73.1	61.5	57.7	50.0	42.3	38.5	38.5	38.5 (111)	NS
	SA®	61	75.4	65.6	49.2	42.6	39.3	36.1	31.1	21.3 (160)	
Secondary implant	proSA®	111	91.0	83.8	73.9	68.5	64.9	63.1	61.3	54.1 (145)	NS
	SA®	193	83.9	77.7	75.1	68.9	64.8	63.2	61.1	56.0 (166)	

(%) Data in parentheses are percentages. *PHH* post-hemorrhagic hydrocephalus, *IAS* idiopathic aqueduct stenosis, *NS* not significant

Regarding the etiology of hydrocephalus (posthemorrhagic HC, idiopathic aqueduct stenosis, Chiari malformation type II), both cohorts showed similar results in terms of survival time and the number of explanted vs. non explanted SA® (*P* = 0.057, *P* = 0.073) or proSA® valves (*P* = 0.724, *P* = 0.472). No significant difference in valve survival rate was observed after the inter-cohort comparison for the same etiological subgroups (*P* = 0.911; *P* = 0.686; *P* = 0.686).

In both cohorts, the mean patient’s age was significantly younger among the explanted valves (*P* < 0.01), and the valve survival rate was considerably lower in the first age subgroup (0–2 years) compared to the other two groups (2–10 and 10–18 years) (*P* < 0.01). Below 2 years of age, mean valve survival time was 40.5 ± 32.4 and 40.9 ± 42.1 months for proSA® and SA®, compared to 69.3 ± 39.2 and 56.7 ± 39.7 in the second group (2–10 years). We observed no significance when comparing proSA® and SA® survival rates for the three age subgroups (*P* = 0.954; *P* = 0.059; *P* = 0.806).

In the SA® cohort, primary implants were associated with significantly more valve explantation and a lower survival rate than the secondary implanted valves (*P* < 0.001). There was no significant difference in valve survival rate among proSA® and SA® for primary (*P* = 0.769) or secondary implants (*P* = 0.208). Age at implantation was significantly younger for primary than secondary implants (1.9 ± 3.8 vs. 6.8 ± 5.3 years; *P* < 0.001). One-year survival rate in primary inserted proSA cohort was 73.1% compared to 75.4% in primary inserted SA cohort. Additionaly, the 5-year survival rate was 42.3% in same proSA cohort vs 39.3% in SA cohort.

### Biomechanical analysis

**Fig. 4:**
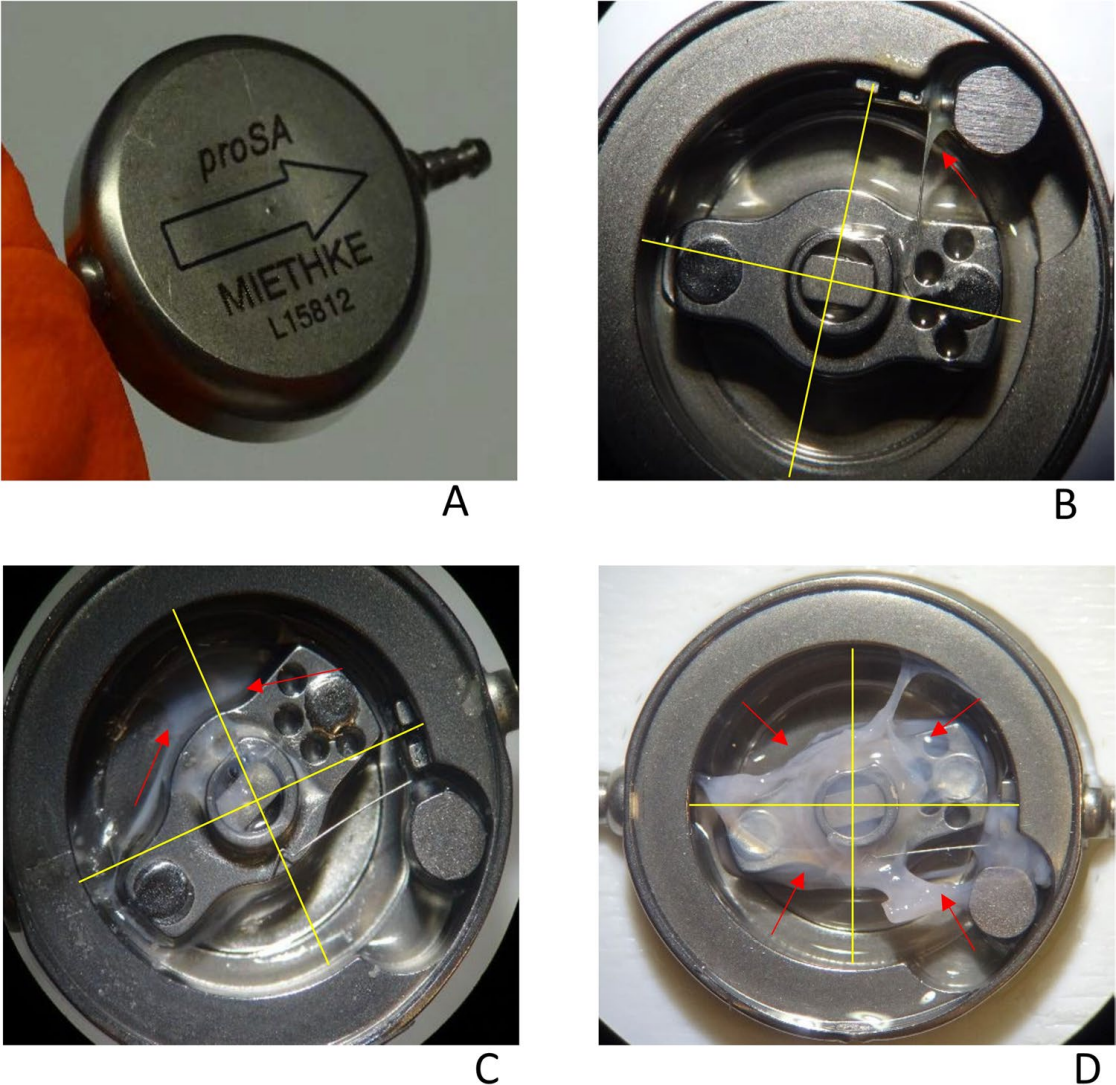
Internal deposition and deformation of the external valve housing. Figure **A** illustrates a severe deformation of the proSA®, while grade I, II, and III deposits can be observed in figures **B**, **C**, and **D**, respectively. Yellow lines divide the valves into quadrants to evaluate the deposit grade (I < 25%, II 25–50%, III > 50%). Red arrows indicate the presence of deposits in the quadrants

Manufacturer’s reports of the biomechanical analysis were available for 41 proSA® and 31 SA® explanted valves. Impossible (*n* = 21) or spontaneous adjustments (*n* = 3) of the pressure settings represented the first cause of valve explantation in the analyzed proSA® valves (58.4%), while overdrainage (35.5%) and suspected SA® dysfunction associated with defective DP valves (38.7%) led to significantly more valve explantation in the other group (*P* < 0.001). The external housing was deformed in 51.2% of the proSA® valves, while presented only scratches in 4 proSA® and 2 SA® valves. Reduced permeability or complete occlusion was documented in 46.3% and 32.3% of the proSA® and SA® valves, respectively (*P* = 0.241). The measurement of the flow resistance performed showed an accelerated flow after the vertical valve orientation in 50.0% of proSA® and 29.2% of SA® valves (*P* = 0.949). Internal deposits were observed in 97.6% and 90.3% of the proSA® and SA® valves. The proSA® internal surface was covered mainly by grade 3 (51.2%) and grade 2 (26.8%) deposits, while grade 1 extents were observed in 19.5% of the cases. The intraoperative CSF examination was utterly unremarkable in both cohorts. (For more details, see Table 4 and Fig. 4.)

**Table 4:** Biomechanical analysis of the explanted valves*

	proSA®	SA®	*P*-value**
Analyzed valves/explanted valves	41/64 (64.0)	31/118 (26.3)	-
Mean age (years)**	3.76 ± 4.455	3.34 ± 4.24	0.684
Mean survival time (months)***	49.22 ± 37.74	47.25 ± 41.87	0.837
Hydrocephalus etiology			0.164
Post-hemorrhagic	21 (51.2)	17 (54.8)	
Idiopathic aqueduct stenosis	8 (19.5)	5 (16.1)	
Chiari malformation type I	0	1 (3.2)	
Chiari malformation type II	9 (22.0)	2 (6.5)	
Postinfectious	1 (2.4)	4 (12.9)	
Arachnoid cyst	1 (2.4)	0	
Unclear	1 (2.4)	1 (3.2)	
Indication for proSA® or SA® valve explantation			** < 0.001**
Gravitational valve adjustment difficulties	24 (58.5)	0	
DP valve adjustment difficulties	4 (9.8)	12 (38.7)	
Overdrainage	1 (2.4)	11 (35.5)	
Underdrainage	5 (12.2)	4 (12.9)	
Catheter-related causes	7 (17.1)	2 (6.5)	
Others	0	2 (6.5)	
Slit ventricle before valve explantation	16 (39.0)	20 (64.5)	0.056
Intraoperative CSF examinations performed (*n*)	28 (68.3)	27 (87.1)	0.093
Intraoperative CSF content*** (reference)			
Protein (< 0.4 g/l)	0.146 ± 0.099	0.189 ± 0.185	0.292
Glucose (49–75 mg/dl)	47.14 ± 8.25	58.23 ± 8.73	0.638
Lactate (1.1–1.8 mmol/l)	1.27 ± 0.36	1.22 ± 0.31	0.541
Leukocytes (< 5/µl)	2.92 ± 5.34	2.04 ± 2.61	0.455
Biomechanical analysis			** < 0.001**
Macroscopic changes in the valve casing			
No deformation nor scratches	16 (39.0)	29 (93.5)	
Deformed	21 (51.2)	0	
Only scratches	4 (9.8)	2 (6.5)	
Valve permeability			0.241
Permeable	22 (53.7)	21 (67.7)	
Reduced permeability	5 (12.2)	5 (16.1)	
Occluded	14 (34.1)	5 (16.1)	
Flow resistance in the vertical position			0.949
Test performed****	18 (43.9)	24 (77.4)	
Within tolerance	9 (50.0)	17 (70.8)	
Below the normal range	9 (50.0)	7 (29.2)	
Adjustability in vitro			**-**
Adjustable	32 (78.0)	-	
Adjustment dysfunction	9 (22.0)	-	
No longer adjustable	7 (17.1)		
Limited adjustment range	2 (4.9)		
Click force and brake function test			**-**
Not performed	7 (17.1)	31 (100)	
Normal braking function	25 (61.0)	-	
Defective braking function	9 (22.0)	-	
The extent of deposition (% of inner valve surface)			-
Absence	1 (2.4)	3 (9.7)	
Grade I (< 25%)	8 (19.5)	-	
Grade II (25–50%)	11 (26.8)	-	
Grade III (> 50%)	21 (51.2)	-	

(%) Data in parenthesis are percentages

^*^Without the early wound complications group

^**^Bold denotes statistical significance

^***^Data are given as mean ± standard deviation

^****^The flow resistance test is performed in cases of suspected valve-related overdrainage and cannot be performed in cases of non-permeable valves

### Clinical and radiological outcomes

In our study, pre- and postoperative clinical assessments were conducted for all implanted valves. The most common clinical manifestations were headache, nausea, increased head circumference, and arrests of head growth. A postoperative improvement was observed for 92.2% and 88.4% of the symptoms and signs in the proSA® and SA® cohorts, respectively. A arrest of the head growth rate for over 3 months in children below 2 years of age was considered an adverse finding of persistent overdrainage. This condition was observed preoperatively in 13 and 11 cases in the proSA® and SA® cohorts, respectively. After surgery, 84.6% of the proSA® cases retained regular head growth rates, compared to 54.5% of the SA®.

A postoperative radiological improvement in the previously documented slit ventricles morphology was observed in 25.4% and 26.9% of the cases in proSA® and SA® cohorts, respectively. In the proSA® cohort, the presence of preoperative slit ventricle was associated with significantly less valve explantation (*P* = 0.037), while no significant difference was observed for the SA® valves based on preoperative radiological findings of overdrainage (*P* = 0.054). This means that proSA® was more efficient in achieving clinical improvement through adjustments sparing operative revisions, while SA® was more likely to require valve revision for clinical overdrainage with corresponding radiological evidence.

## Discussion

### Summary of the results

This study is the first one to compare the clinical benefits and survival rates of programmable gravitational valves with their fixed gravitational valve predecessors in pediatric patients with hydrocephalus. Previous authors have documented experiences with both valve types [2, 4, 9, 11, 16, 18, 25, 30] separately. The 1- and 5-year valve survival rates were 87.6% and 60.6% for proSA®, and 81.9% and 58.7% for SA®, respectively. Young age at implantation (< 2 years) was a risk factor for valve failure in both cohorts (*P* < 0.001). However, no significant difference was observed in any age or etiological subgroup. The indications for valve explantation were significantly different between the two groups (*P* < 0.001), with overdrainage being the main reason for SA® explantation (41.5% vs. 3.1%) and adjustment difficulties (45.3%) in the proSA® cohort (*P* < 0.001). Programmable valves were more effective in controlling overdrainage than their predecessors, but they were limited by an increased susceptibility to mechanical malfunctions. The etiology of hydrocephalus, CSF protein content, and the number of valve adjustments did not contribute significantly to valve explantation, according to our study.

### Programmable vs. fixed gravitational valves

Gravitation-assisted adjustable DP valves (e.g., proGAV®) have been widely used in the last two decades, showing promising results in preventing chronic overdrainage in young patients [6, 11, 25, 28, 29]. The long-term valve survival rates of gravitation-assisted adjustable DP valves were evaluated by Gebert et al. [9] in 93 infants, reporting valve survival rates of 77.8% and 58.2% at 12 and 85 months, respectively. Our study assessed the gravitational valve (SA®) survival rate separately, obtaining comparable results with 1- and 7-year valve survival of 87.0% and 57.3%. Recent studies assessed the use of proSA® whether with fixed DP valves (miniNAV®) in infants [4] or in adolescent and adult patients with a complex shunt history [18, 30] and reported 12 and 24 months valve survival rates of 91% and 90%, respectively. Our study observed a 1-year valve survival rate of 89.6% for proSA®, which also aligns with the prospective multicentric PROSAIKA study (89%) [16]. Besides the current emphasis on adjustable DP valves, Sokratous et al. [27] showed positive results with 235 fixed gravitational valves (paediGAV® Miethke) implanted mainly in pediatric patients, encouraging their use before others in case of uncomplicated hydrocephalus. Therefore, selecting the optimal gravitational valve remains challenging due to the lack of comparative studies and longer follow-up for proSA® valves.

### Age and etiology

Premature infants and young patients with intraventricular hemorrhage represent a vulnerable group with a higher risk for shunt failure and adverse neurodevelopmental and growth outcomes [1, 5, 19, 24, 29]. In our study, young age proved to severely affect valve survival in both cohorts (*P* < 0.001), while posthemorrhagic etiology of hydrocephalus showed no statistical significance. Similar results were previously reported by the prospective multicentric study of Riva-Cambrin et al. [24], observing that only age (< 6 months) and no etiology affected shunt survival. We observed a higher valve survival rate for secondary implantations in both cohorts; however, this finding might be misleading given the younger age of patients at the insertion of primary implants ( 1.9 ± 3.8 vs. 6.8 ± 5.3 years; *P* < 0.001).

Based on our findings, it is justifiable to use a fixed gravitational valve as the primary implant for infants and young children (below 2 years of age) as during the early months after birth, these patients typically lie down and are at a lower risk of overdrainage. Therefore, the benefits of programmable valves would be limited, and the comparable survival rate would not warrant the higher cost and the risk of further revisions. However, in older children (between 2 and 10 years), the average survival time increases significantly, by approximately twofold (103.0 ± 7.3 vs. 51.2 ± 6.9 months). Therefore, in selected cases where symptomatic patients require treatment flexibility throughout the child’s growth, substituting SA® with proSA® valves would help achieve better clinical outcome and avoid the long-term sequelae of overdrainage.

### Valve adjustment and overdrainage

Pressure settings were adjusted in 67.2% of the proSA® valves, allowing better control of overdrainage, representing the main indication (74.5%). Compared to the SA® group, the proSA® valve was associated with more remarkable clinical improvement, particularly regarding overdrainage-related complications, e.g., head growth arrests. Nevertheless, in addressing slit ventricular morphology, proSA® showed similar outcomes as its precursor (25.4% vs. 26.9%). The discrepancy between clinical and radiological improvement was priorly observed [30] and attributed to the need for longer time to witness ventricular morphology changes once the optimal pressure setting for the proSA® valve is reached [13, 18, 31]. As reported by Hall et al., the persistence of slit ventricles despite satisfactory clinical improvement suggests that ventricular metrics may not be accurate parameters for evaluating overdrainage correction [12].

### Biomechanical analysis

Conducting a biomechanical analysis is a practical way to thoroughly evaluate the mechanical dysfunctions that result in valve revisions [3]. Internal depositions of protein and cellular materials in titanium-based valves were previously described by Ludwig et al. [17] and were observed in 97.6% and 90.3% of the analyzed proSA® and SA® valves. In proSA® valves, they were associated with more valve occlusions (34.1% vs. 16.1%) and other mechanical defects affecting the adjustment (22.0%) and braking system (22.0%). These findings suggest that the adjustability of the gravitational valves implies an increased vulnerability to damage due to protein deposits. Severe deformation of the valve casing which was observed only in the proSA® series (39.0%, *P* < 0.001) is probably due to multiple trials of failed adjustments.

### Limitations

The findings of this study should be interpreted within the scope of its limitations. During the long observation period, multiple surgeons performed the interventions. Given the retrospective nature of the data, the cause of shunt failure assessment may be challenging and exposed to the risk of bias. The number of cases differed significantly between the two cohorts, as the type and age of the DP valve implanted. Gravitational valves were independently tested during DP valve explantation; however, uncertain intraoperative findings may have implied their precautionary explantations, thus underestimating relative survival. Our analysis did not address the potential impact of the DP unit on the performance of the gravitational unit in a dual valve system. It is important to note that the series examined in this study primarily consisted of secondary implanted valves, resulting in an average age that is higher compared to other studies that focused on the primary use of gravitational valves in pediatric patients. Biomechanical analysis of valves was conducted for only part of the observation period; hence, the number of evaluated explants was limited. A more accurate quantitative assessment of the deposits would be needed to better define their extent.

## Conclusions

The proSA® and SA® valves showed almost similar long-term results and survival rates. The programmability may be beneficial regarding better control of symptoms and should be used to address complex hydrocephalus cases with symptoms and signs of chronic overdrainage. Young age at implantation still poses the leading risk factor for valve explantation. Therefore, considering their use in selected patients after 2 years of age could increase the survival time and maximize the benefits of the more expensive programmable gravitational valves. However, due to the elevated incidence of mechanical dysfunctions, the proSA® valve did not reduce the need for shunt revisions compared to its precursor. The presence of internal deposits might affect the adjustment system representing a major limitation of these sophisticated devices.

## Data Availability

The data presented in this study are available on request from the corresponding author.
